# Cytokine and chemokine multiplex analysis-based exploration for potential treatment and prognostic prediction in large-vessel vasculitis: A preliminary observational study

**DOI:** 10.3389/fimmu.2022.1066916

**Published:** 2022-11-23

**Authors:** Nobuya Abe, Michihiro Kono, Michihito Kono, Takayuki Katsuyama, Kazumasa Ohmura, Taiki Sato, Kohei Karino, Yuichiro Fujieda, Masaru Kato, Rie Hasebe, Masaaki Murakami, Tatsuya Atsumi

**Affiliations:** ^1^ Department of Rheumatology, Endocrinology and Nephrology, Faculty of Medicine and Graduate School of Medicine, Hokkaido University, Sapporo, Japan; ^2^ Division of Molecular Psychoimmunology, Institute for Genetic Medicine, Graduate School of Medicine, Hokkaido University, Sapporo, Japan; ^3^ Department of Nephrology, Rheumatology, Endocrinology and Metabolism, Graduate School of Medicine, Okayama University, Okayama, Japan; ^4^ Centre for Infectious Cancers, Institute for Genetic Medicine, Hokkaido University, Sapporo, Japan

**Keywords:** large vessel vasculitis, Takayasu arteritis, giant cell arteritis, proteomics, cytokine, chemokine, clustering, Janus-kinase inhibitor

## Abstract

Large-vessel vasculitis (LVV) is subclassified into two phenotypes; Takayasu arteritis and giant cell arteritis. Although the pathogenesis of LVV is not fully established, IL-6−IL-17 axis and IL-12−IFN-γ axis play critical roles in the disease development. We aimed to clarify the association between the disease state and cytokine/chemokine levels, to assess disease course as prognosis and to predict regulators in patients with LVV using the blood profiles of multiple cytokines/chemokines. This retrospective analysis comprised 35 LVV patients whose blood were collected, and multiplex cytokine/chemokine analysis with 28 analytes was performed. The differences of cytokines/chemokines corresponding disease status, upstream regulator analysis, pathway analysis and cluster analysis were conducted using the cytokines/chemokines profile. Relapse-free survival rate was calculated with Kaplan-Meier analysis in the classified clusters. In the robust analysis, IL-4, CCL2/MCP-1, TNFSF13/APRIL, TNFSF13B/BAFF, CHI3L1 and VEGF-A levels were significantly changed after treatment. Untreated LVV patients demonstrated activation of NFκB-related molecules and these patients are potentially treated with JAK/STAT inhibitors, anti-TNF-α inhibitors and IL-6 inhibitors. Cluster analysis in active LVV patients revealed two clusters including one with high blood levels of IL-1β, IL-6, IL-17, IL-23 and CCL20/MIP-3. A subgroup of the LVV patients showed activated IL-17 signature with high relapse frequency, and JAK/TyK2 inhibitors and IFN-γ inhibitors were detected as potentially upstream inhibitors. Blood cytokine/chemokine profiles would be useful for prediction of relapse and potentially contributes to establish therapeutic strategy as precision medicine in LVV patients.

## Introduction

Large-vessel vasculitis (LVV) is a class of the vasculitides, mostly affecting large arteries, and is subclassified into Takayasu arteritis (TAK) and giant cell arteritis (GCA). There are some differences in the predilection site; TAK often shows pan-aortitis and GCA affects the branches of the carotid arteries, including the superficial temporal artery. Meanwhile, previous reports showed substantial similarities in the distribution of arterial lesions between TAK and GCA, suggesting that TAK and GCA could share a disease spectrum to some extent (1–3). Although the pathogenesis of LVV is not entirely clear, the biopsied arteries from patients with TAK and GCA share a lot of pathologic features (4), providing the perspective that TAK and GCA would represent similar pathophysiology. One of the most fundamental pathophysiological profiles in LVV is related to the dysregulated interaction of vascular endothelial cells and immune cells (5). Endogenous vascular dendritic cells recruit activated CD4^+^ T cells, which include interferon-γ (IFN−γ)–secreting T helper 1 (Th1) cells and interleukin−17 (IL-17)-secreting T cells (Th17). A particular set of cytokines is required for the differentiation of naïve CD4^+^ T cells into Th1/Th17. A previous report demonstrated that patients with GCA showed an elevated serum IL-6 level, which has a vital role in Th17 cell differentiation (6). Also, IFN-γ, mainly produced by IL-12-inducible Th1 cells, is associated with the pathogenesis of LVV *via* vascular smooth muscle proliferation and endothelial cell induction for the proinflammatory phenotype (7). A study revealed elevated plasma levels of IFN-γ in patients with GCA (8). Thus, multiple cytokine axes centring on the IL-6−IL-17 axis and the IL-12−IFN-γ axis play prominent roles in the development of LVV.

Recently, proteomic analysis was widely used to understand complex biological networks in various diseases. There are proteomic studies for the pathophysiological clustering by serum cytokine/chemokine profiles in the patients with some diseases such as Sjögren’s syndrome and psoriatic arthritis (9, 10). In LVV, a few proteomic studies analysing serum cytokines/chemokines were reported, and the analysis results are useful for clarifying pathogenic pathways associated with TAK and evaluating the disease activity of TAK (11–13). However, there has been no study analysing serum cytokine/chemokine profiles to predict the clinical courses of patients with LVV. Thus, the present study aimed to explore the immunological/inflammatory profiles of LVV patients using multiplex cytokine/chemokine analysis that reflects the clinical outcome as a prognosis.

## Method

### Patients and data extraction

We retrospectively identified 35 LVV patients whose sera were preserved at the laboratories of Hokkaido University or Okayama University. They were diagnosed with either TAK or GCA in accordance with The American College of Rheumatology 1990 classification criteria from January 2008 to March 2021 at Hokkaido University Hospital or Okayama University Hospital. Among these patients, 15 TAK patients and 20 GCA patients were included. The patients’ baseline data including demographics, laboratory findings, initial therapeutic regimens, and clinical courses were extracted. Disease activity of LVV is defined according to 2018 the EULAR recommendations (14). In brief, the definition of active LVV includes (1); the presence of typical signs and symptoms of active LVV such as new-onset or worsening of limb claudication, constitutional symptoms, myalgia, arthritis, abdominal pain, stroke, angina and paresis of extremities in TAK, and new-onset of persistent localized headache often in the temporal area, constitutional symptoms, jaw claudication, and acute visual symptoms including amaurosis fugax, and polymyalgia rheumatica in GCA (2); the presence of current activity on imaging or biopsy, ischemic complications, or persistently elevated inflammatory markers. An inactive disease is defined as the absence of all the clinical signs and symptoms attributable to active LVV and normalization of inflammatory markers. We collected the sera of the patients with active disease without treatment, or inactive disease irrespective of the timing of the therapeutic course. We described the event rates referring to initial relapse. Ethical aspects of this study were approved by the Institutional Review Board of Hokkaido University Hospital (reference number: 020-0042) and that of Okayama University. The present study complied with the Declaration of Helsinki. We obtained the written informed consents for this study and publication from all the patients included in this study.

### Multiplex cytokine/chemokine analysis

Multiplex cytokine/chemokine beads assays were performed using sera or plasma of the patients and a Luminex^®^ (Performance) Assay (R&D Systems, Minneapolis, MN, USA), and analysed with a Luminex^®^ 200 xPONENT^®^ System (Merck KGaA, Darmstadt, Germany). The cytokines and chemokines measured by the multiplex beads assay included tumour necrosis factor α (TNFα), IFN-γ, IL-1β, IL-2, IL-4, IL-5, IL-6, IL-8/C-X-C motif chemokine ligand (CXCL) 8, IL-10, IL-12p70, IL-13, IL-17, IL-18, IL-23, IL-1 receptor-like 1/ST2, CXCL1/growth-related oncogene α, CXCL13/B lymphocyte chemoattractant, C-C motif chemokine ligand (CCL) 2/monocyte chemotactic protein-1 (MCP-1), CCL3/macrophage inflammatory protein-1α (MIP-1α), CCL5/Regulated on Activation, Normal T cell Expressed and Secreted (RANTES), CCL7/MCP-3, CCL20/MIP-3, TNF ligand superfamily member 13 (TNFSF13)/A proliferation-inducing ligand (APRIL), TNFSF13B/B cell activating factor belonging to the tumour necrosis factor family (BAFF), Chitinase-3-like-1 (CHI3L1), epidermal growth factor, fibroblast growth factor 23 (FGF-23), and vascular endothelial growth factor (VEGF)-A. The sera of the patients were collected at the active disease and the inactive disease. The collected serum and plasma were centrifuged at 3000 rpm at 4°C for 15 minutes, and the supernatant was stored at -80°C until use.

### Proteomic analysis and cluster analysis

The ratio of the average blood cytokine/chemokine level between the groups was used for the following subsequent analysis. Upstream regulators analysis and pathway analysis were performed with blood cytokines/chemokines profile using Ingenuity Pathway Analysis (IPA) (QIAGEN, Hilden, Germany) (15). Clusters of the patients were identified by blood cytokine/chemokine levels. We performed hierarchical clustering analysis by applying an unsupervised agglomerative method with complete-linkage method. Results were shown as dendrograms with heatmaps using standardized values of cytokines and chemokines *via* scaling the range from 0 to 1. The optimal number of clusters was determined with reference to the silhouette plot. Cluster analysis was performed by JMP^®^ Pro 14 (SAS Institute Inc., Cary, NC, USA). Using the clustering data, we analyse the difference between the clusters regarding cytokine/chemokine profiles and the patients’ characteristics and clinical outcomes in LVV to discover disease subsets of LVV.

### Statistical analysis

We used the Mann-Whitney U test, and Pearson’s chi-squared test to compare the unpaired values of continuous variables, and proportions of categorical variables between the groups, respectively. Wilcoxon signed-rank test was used to compare the paired values. Kaplan–Meier survival curves were produced to illustrate the prognostic value of the clusters using the log-rank test. We used JMP^®^ Pro 14 (SAS Institute Inc., Cary, NC, USA) for all analyses. When the p-value was under 0.05, the results demonstrated statistical significance. All statistical tests were two-sided.

## Results

### Activated molecules of NFκB-related pathway in the untreated patients with LVV

**Figure 1:**
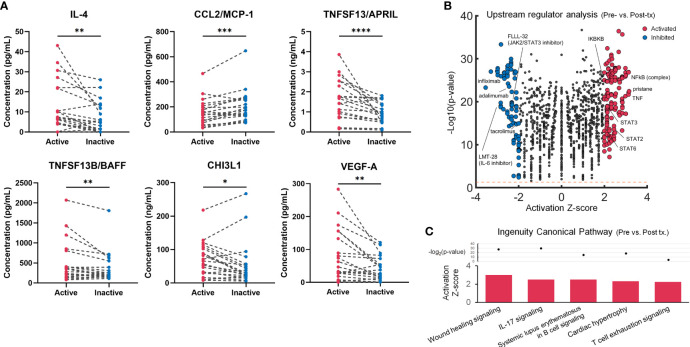
Multiplex blood cytokine/chemokine analysis results in the untreated and treated patients with large-vessel vasculitis (LVV). **(A)** Twenty-one blood samples from 21 patients with LVV in active or inactive state were examined using multiplex cytokines/chemokines analysis. A dot plot with a line shows individual cases. *p < 0.05, **p < 0.01, ***p < 0.001, ****p < 0.0001, Wilcoxon signed-rank test. **(B)** Volcano plot displaying significant regulators predicted by Ingenuity Pathway Analysis for untreated patients with LVV compared to post-treated patients. The y-axis corresponds to the value of -log_10_(p-value), and the x-axis displays the activation Z-score value. The red dots represent the predictably activated regulators or the blue dots inhibited. Positive x-values represent probable activation and negative probable inhibition. The Orange horizontal line denotes p-value of 0.05. **(C)** Ingenuity canonical pathways with the prediction of its activated pathway in pre-treated vs. post-treated patients with LVV.

We identified analysis objects as 35 samples with active disease and 21 paired samples with inactive disease. Baseline characteristics were summarized in **Supplementary Table S1**. The median follow-up duration of all the LVV patients was 30 months. During the follow-up period, the overall rate for initial relapse was 20%. The clinical manifestations, affected vessels, and imaging analysis results were available in **Supplementary Table S2**. All of TAK patients and 75% of GCA patients demonstrated LVV involvements. The multiplex cytokine/chemokine analysis revealed that several analytes including IL-4, CCL2/MCP-1, TNFSF13/APRIL, TNFSF13B/BAFF, CHI3L1 and VEGF-A levels were significantly changed in inactive LVV compared with active disease (**Figure 1A**; **Supplementary Figure S1**). In a sensitivity analysis for TAK and GCA patients, IL-4, TNFSF13/APRIL and VEGF-A showed similar trends to robust analysis. However, CCL2/MCP-1, TNFSF13B/BAFF and CHI3L1 levels differed between TAK and GCA (**Supplementary Figure S2**). The upstream regulator analysis comparing the cytokine/chemokine profiles in the pre-treatment with those in post-treatment predicted 227 upstream regulators with statistical significance. These included activated molecules of NFκB-related pathway and potential inhibitors including anti-TNF antibodies, IL-6 inhibitors and JAK-STAT inhibitors (**Figure 1B**), a part of which is clinically available (e.g., infliximab and adalimumab). The pathway analysis demonstrated activating pathways related to wound healing, cardiac hypertrophy, immune cells, and IL-17 signalling in the untreated patients with LVV (**Figure 1C**).

### Frequent relapse observed in the LVV patients with activated IL-17 signature

**Figure 2:**
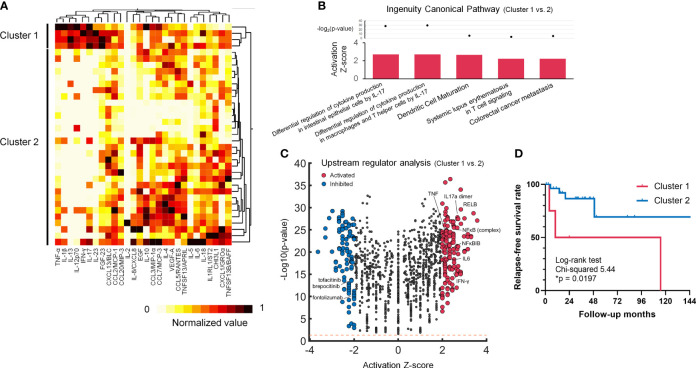
Cluster analysis in untreated LVV patients, revealing the activating IL-17 phenotype with frequent relapse. **(A)** Cluster heatmap of blood 28 cytokines/chemokines data of the normalized concentration values. **(B)** Volcano plot displaying significant regulators predicted by Ingenuity Pathway Analysis for Cluster 1 LVV patients in comparison with Cluster 2. The y-axis corresponds to the value of -log_10_(p-value), and the x-axis displays the activation Z-score value. The red dots represent the predictably activated regulators or the blue dots inhibited. Positive x-values represent probable activation and negative probable inhibition. The Orange horizontal line denotes p-value of 0.05. **(C)** Ingenuity canonical pathways with the prediction of its activated pathway in Cluster 1 vs. Cluster 2 LVV patients. **(D)** Kaplan-Meier survival curve regarding relapse free survival rate during the time from the initial diagnosis to the first documented disease relapse. The LVV patients in Cluster 1 (red) demonstrated a higher relapse survival rate (*p < 0.05) than those in Cluster 2 (blue) using Log-rank test. The ticks indicate censored data.

Although IL-17 signalling activation was suggested in the pre-treated LVV patients, Th17-associated cytokines/chemokines such as IL-1β, IL-6, IL-17, IL-23 and CCL20/MIP-3 showed highly variant levels among the untreated LVV patients (**Supplementary Figure S1**). It was possible to subgroup the LVV patients according to blood cytokine/chemokine profiles. We adopted hierarchical clustering analysis in the 35 active LVV patients, classified into two subgroups (**Figure 2A**). Some analytes including TNF-α, IFN-γ, IL-1β, IL-12p70, IL-13, IL-17, IL-23 and FGF-23 showed the high fraction of variation (R^2^ value) explained by the clustering (**Supplementary Table S3**). The characteristics between the clusters of the active LVV patients are summarized in **Supplementary Table S4**. All the LVV patients included in Cluster 1 were diagnosed with TAK and younger than those in Cluster 2. Using the ratio of measured cytokine/chemokine levels in active LVV patients of Cluster 1 to those of Cluster 2, IPA revealed that IL-17-induced differential regulation of cytokine production was significantly upregulated in Cluster 1 (**Figure 2B**). In upstream regulator analysis, 262 molecules were predicted as significant regulators in Cluster 1 (**Figure 2C**). The upstream activators included TNF, IL-17a dimer, IL-6, RELB, and NFκB. The inhibitors included tofacitinib and brepocitinib, JAK/Tyk2-STAT inhibitors, and fontolizumab, a humanized monoclonal antibody for IFN-γ. Furthermore, the patients in Cluster 1 demonstrated a significantly higher relapse rate than those in Cluster 2 (75% vs. 14%, p = 0.023). Kaplan-Meier survival analysis also revealed a significantly lower relapse-free survival rate in the LVV patients of Cluster 1 than Cluster 2 (**Figure 2D**). In subgroup analysis limited to TAK, the patients in Cluster 1 also showed a higher tendency of relapse than those in Cluster 2 (75% vs. 25%, p = 0.074). In contrast, baseline laboratory findings at diagnosis, and therapeutic strategy were similar between the clusters (**Supplementary Table S4**).

## Discussion

The current study showed that multiplex cytokines/chemokines profiles suggested activation of NFκB-related molecules in untreated patients with LVV and potential therapeutic agents inhibiting TNF-α, IL-6 or JAK-STAT for active LVV. The profiles also revealed a subgroup of LVV patients showing activated IL-17 signature and high relapse frequency, which is potentially inhibited by JAK/TyK2 inhibitors.

The upstream regulator analysis in pre-treated and post-treated LVV patients showed activated NFκB molecules and potential inhibitors such as JAK/Tyk2-STAT inhibitors, which were also suggested in the analysis of clusters (16, 17). A recent study using peripheral CD4^+^ and CD8^+^ T cells from patients with TAK highlighted the transcriptional enrichment of JAK-STAT signalling and IFN signature, and tofacitinib could significantly decrease Th1/Th17 cells, which secrete IFN-γ/IL-17 (18). A basic study using immunodeficient mice engrafted by human arteries and reconstituted with T cells from GCA patients demonstrated that tofacitinib effectively suppressed tissue-resident memory T cells with decreasing IFN-γ and IL-17 (19). The signalling pathway of Th1/Th17 has been reported to play roles in the pathogenesis of LVV. Consistently, Th1 and Th17 cells are detected in the vasculitic lesions of LVV (20). The studies, including ours suggest the importance of NFκB-related molecules and JAK-STAT signalling for the development of LVV.

Furthermore, in general, Th17 cells are resistant to glucocorticoid-induced apoptosis (21), suggesting that activated IL-17 signalling is potentially resistant to conventional immunosuppressive therapy including glucocorticoids, leading to persistent vasculitis accompanying frequent relapses in a part of LVV patients with IL-17 activated signature. Inhibiting the IL-17 signature would be an effective treatment strategy for refractory LVV patients. Many recent case reports have demonstrated that JAK inhibitors are effective for LVV refractory to general immunosuppressants and biologics, including anti-TNF-α and anti-IL-6 therapy (16, 17). Thus, JAK/Tyk2-STAT pathway would be associated with LVV development and therapeutic resistance, becoming a promising therapeutic target.

We acknowledge several limitations in this study. First, this is a retrospective study, which may lead to statistical underpower by the small sample size. In this study, we analysed cytokines and chemokines in patients with TAK and GCA as LVV, one disease. Although these entities do not show completely same profiles, our study combining TAK and GCA could reveal that a part of TAK patients showed similar cytokine and chemokine profiles to GCA patients. Our results suggest that disease classification of LVV by cytokine and chemokine profiles would be more useful for establishing pathogenesis and therapeutic strategy than by conventional criteria for TAK and GCA. Second, the prognosis is dependent on the severity of the disease at the time of assessment and on the response to therapy. Sensitivity analysis for TAK and GCA is required to clarify the similarities and differences in pathogenesis therapeutic responses between these diseases. We require further multi-centre study using a large number of LVV patients to assess the value of the classification with the clinical entity by cluster analysis with proteomics.

In conclusion, we demonstrated that untreated LVV patients demonstrated NFκB activation, which potentially treated by the inhibition of TNF-α, IL-6 or JAK-STAT. Moreover, some patients with LVV had an activated IL-17 signature in serum cytokine/chemokine profiles, leading to a higher relapse rate than the patients without the signature. Blood cytokine/chemokine profile would be useful for risk stratification of relapse in LVV. Although further studies are needed, multiplex cytokine/chemokine analysis potentially contributes to the establishment of pathogenesis and therapeutic strategy for precision medicine in LVV patients.

## Data availability statement

The original contributions presented in the study are included in the article/**Supplementary Material**. Further inquiries can be directed to the corresponding author.

## Ethics statement

The studies involving human participants were reviewed and approved by the Institutional Review Board of Hokkaido University Hospital. The patients/participants provided their written informed consent to participate in this study.

## Author contributions

NA: Conceptualization, Investigation, Methodology, Software, Formal analysis, Writing – original draft. MK (2nd author): Conceptualization, Investigation, Resources, Writing – original draft. MK (3rd author): Conceptualization, Methodology, Writing – original draft, Writing – review & editing. TK: Investigation, Resources, Writing – original draft. KO, TS, KK: Investigation, Resources. YF, MaK: Writing – review and editing. RH, MM: Methodology, Software. TA: Writing – review and editing, Supervision. All authors contributed to the article and approved the submitted version.

## Acknowledgments

We thank Ms. Yumiko Kaneko, Ms. Yui Ishikura, Mr. Yusuke Fijita and Ms. Chiemi Nakayama for their technical support, and Mses. Shikishi Chida and Satomi Fukumoto for their secretary support. We also appreciate Prof. Jun Wada, Department of Nephrology, Rheumatology, Endocrinology and Metabolism, Graduate School of Medicine, Okayama University, Okayama, Japan, for constructive discussion.

## Conflict of interest

MK (3rd author) reports grants from GlaxoSmithKline plc, Mitsubishi Tanabe, Astellas, Sanofi, Taisho Pharmaceutical, NIPPON SHINYAKU CO., LTD., Taiju Life Social Welfare Foundation, Kowa CO. Ltd., Terumo Corporation, KYOCERA Corporation, CHUGAI PHARMACEUTICAL CO., LTD., MOCHIDA PHARMACEUTICAL CO., LTD., LOTTE CO., LTD., Hitachi, Ltd., Takeda Pharmaceutical CO. Ltd., and YAMAZAKI BAKING CO., LTD., outside the submitted work. MaK has received research grants from AbbVie, Actelion, and GlaxoSmithKline, and speaking fees from Eli Lilly. TA reports grants from Astellas, Takeda Pharmaceutical, Mitsubishi Tanabe, Chugai Pharmaceutical, Daiichi Sankyo, Otsuka Pharmaceutical, Pfizer, and Alexion, and personal fees from Mitsubishi Tanabe, Chugai Pharmaceutical, Astellas, Takeda Pharmaceutical, Pfizer, AbbVie, Eisai, Daiichi Sankyo, Bristol-Myers Squibb, UCB Japan, Eli Lilly Japan, AstraZeneca, MEDICAL & BIOLOGICAL LABORATORIES, ONO PHARMACEUTICAL, Novartis, and Nippon Boehringer Ingelheim, outside the submitted work.

The remaining authors declare that the research was conducted in the absence of any commercial or financial relationships that could be construed as a potential conflict of interest.

## Publisher’s note

All claims expressed in this article are solely those of the authors and do not necessarily represent those of their affiliated organizations, or those of the publisher, the editors and the reviewers. Any product that may be evaluated in this article, or claim that may be made by its manufacturer, is not guaranteed or endorsed by the publisher.
