# Supplementary material for: Cytokine and chemokine multiplex analysis-based exploration for potential treatment and prognostic prediction in large-vessel vasculitis: A preliminary observational study
Source: Front Immunol. 2022 Nov 23;13:1066916. doi: 10.3389/fimmu.2022.1066916 (PMC9727250; doi:10.3389/fimmu.2022.1066916)
Supplement: Supplementary file 1 [file DataSheet_1.docx]

***Supplementary material***

| **Supplementary Table 1.** Characteristics of the patients included in this study | | | | |
| --- | --- | --- | --- | --- |
|  |  |  |  |  |
|  | All patients (n = 35) |  |  |  |
| Age at disease onset, year | 61 [31−69] |  |  |  |
| Sex Female | 22 (63%) |  |  |  |
| Body mass index, kg/m^2^ | 21.1 [19.2-22.9] |  |  |  |
| Smoking | 13 (37%) |  |  |  |
|  |  |  |  |  |
| Disease classification |  |  |  |  |
| - Takayasu arteritis | 16 (46%) |  |  |  |
| - Giant cell arteritis | 19 (54%) |  |  |  |
|  |  |  |  |  |
| Follow-up duration, month | 30 [13-45] |  |  |  |
| Relapse | 7 (21%) |  |  |  |
| Death | 0 |  |  |  |
| C-reactive protein at diagnosis, mg/L | 63.3 [19.7-86.3] |  |  |  |
|  |  |  |  |  |
| Initial therapeutic regimen |  |  |  |  |
| - Prednisolone, mg/kg | 0.77 [0.63-0.93] |  |  |  |
| - Immunosuppressants | 12 (34%) |  |  |  |
| Cyclosporine | 1 (3%) |  |  |  |
| Azathioprine | 4 (13%) |  |  |  |
| Methotrexate | 9 (30%) |  |  |  |
| Tacrolimus | 4 (13%) |  |  |  |
| Biologics | 8 (23%) |  |  |  |
| - Infliximab | 1 |  |  |  |
| - Tocilizumab | 7 |  |  |  |
|  |  |  |  |  |

Values are presented as n (%) or median [IQR].

| **Supplementary Table 2.** Clinical manifestations and affected lesions of the patients included in this study | | | |
| --- | --- | --- | --- |
|  |  |  |  |
|  | All patients (n = 35) |  |  |
| **Clinical manifestations** |  |  |  |
| Fever | 17 (49%) |  |  |
| Weight loss (>1.0 kg) | 14 (40%) |  |  |
| Headache | 17 (49%) |  |  |
| Visual impairment | 10 (29%) |  |  |
| Dizziness | 7 (20%) |  |  |
| Claudication | 4 (11%) |  |  |
| Syncope | 1 (2.9%) |  |  |
| Hypertension | 8 (23%) |  |  |
| Pulse deficit | 7 (20%) |  |  |
| Differences in blood pressure | 7 (20%) |  |  |
| Bruit | 6 (17%) |  |  |
| Arthralgia and myalgia | 7 (20%) |  |  |
| Rash | 3 (9%) |  |  |
| Abdominal pain | 3 (9%) |  |  |
| Carotidynia | 6 (17%) |  |  |
| Cardiac failure | 1 (2.9%) |  |  |
| Stroke | 5 (14%) |  |  |
|  |  |  |  |
| **Affected arteries** |  |  |  |
| Aorta | 20 (57%) |  |  |
| Brachiocephalic artery | 18 (51%) |  |  |
| Pulmonary artery | 1 (2.9%) |  |  |
| Thoracic aorta | 13 (37%) |  |  |
| Right subclavian artery | 14 (40%) |  |  |
| Left subclavian artery | 21 (60%) |  |  |
| Right carotid artery | 17 (49%) |  |  |
| Left carotid artery | 19 (54%) |  |  |
| Vertebral artery | 6 (18%) |  |  |
| Abdominal aorta | 13 (37%) |  |  |
| Superior mesenteric artery | 0 |  |  |
| Renal artery | 2 (5.7%) |  |  |
| Iliac artery | 8 (22%) |  |  |
|  |  |  |  |
| **Imaging** |  |  |  |
| Stenosis | 16 (46%) |  |  |
| Dilatation | 1 (2.9%) |  |  |
| Aortic regurgitation | 2 (5.7%) |  |  |
| Bowel ischemia | 1 (2.9%) |  |  |
|  |  |  |  |
|  |  |  |  |
| Values are presented as n (%) or median [IQR]. | |  |  |

| **Supplementary Table 3.** Fraction of variation in cytokine/chemokine levels explained by the clusters of the patients with active large-vessel vasculitis | |
| --- | --- |
|  |  |
|  | R^2^ value |
| TNF-α | 0.5825 |
| IFN-γ | 0.6998 |
| IL-1β | 0.5109 |
| IL-2 | 0.0038 |
| IL-4 | 0.0220 |
| IL-5 | 0.0046 |
| IL-6 | 0.0115 |
| IL-8/CXCL8 | 0.0080 |
| IL-10 | 0.0020 |
| IL-12p70 | 0.7283 |
| IL-13 | 0.9056 |
| IL-17 | 0.6134 |
| IL-18 | 0.0256 |
| IL-23 | 0.6643 |
| IL1RL1/ST2 | 0.0097 |
| CXCL1/GRO alpha | 0.0028 |
| CXCL13/BLC | 0.0739 |
| CCL2/MCP-1 | 0.0016 |
| CCL3/MIP-1 alpha | 0.0395 |
| CCL5/RANTES | 0.0057 |
| CCL7/MCP-3 | 0.0427 |
| CCL20/MIP-3 | 0.0142 |
| TNFSF13/APRIL | 0.0046 |
| TNFSF13B/BAFF | 0.0012 |
| CHI3L1 | 0.0560 |
| EGF | 0.0022 |
| FGF-23 | 0.6625 |
| VEGF-A | 0.0035 |
|  |  |

Abbreviations: TNF, tumour necrosis factor; IFN, Interferon; IL, interleukin; N.A., not applicable; CXCL, CXC chemokine ligand; IL1RL1, IL-1 receptor like 1; GRO, growth-related oncogene; BLC, B lymphocyte chemoattractant; CCL, CC chemokine ligand; MCP, monocyte chemotactic protein; MIP, macrophage inflammatory protein; RANTES, Regulated on Activation, Normal T cell Expressed and Secreted; TNFSF, TNF ligand superfamily member; APRIL, A proliferation-inducing ligand; BAFF, B cell activating factor belonging to the tumour necrosis factor family; CHI3L1, Chitinase-3-like-1; EGF, epidermal growth factor; FGF, fibroblast growth factor; VEGF, vascular endothelial growth factor-A

| **Supplementary Table 4.** Baseline characteristics of the clusters among the patients with active large-vessel vasculitis | | | | |  |  |
| --- | --- | --- | --- | --- | --- | --- |
|  |  |  |  |  |  |  |
|  | Pre-treated patients |  | Cluster 1 | Cluster 2 |  | p-value |
|  | (n = 35) |  | (n = 4) | (n = 31) |  |  |
| **Demographics** |  |  |  |  |  |  |
| Age at disease onset, year | 61 [31-69] |  | 18 [17-26] | 62 [42-71] |  | 0.006 |
| Sex, woman | 22 (63%) |  | 3 (75%) | 19 (61%) |  | 0.59 |
|  |  |  |  |  |  |  |
| **Disease classification and state** |  |  |  |  |  |  |
| TAK | 16 (46%) |  | 4 (100%) | 12 (39%) |  | 0.021 |
| GCA | 19 (54%) |  | 0 | 19 (61%) |  | 0.021 |
|  |  |  |  |  |  |  |
| - ITAS2010 of the patients with TAK |  |  | 3 [1-7] | 4 [2-6] |  | 0.71 |
| - Angiographic classification of TAK |  |  |  |  |  |  |
| Type I |  |  | 1 (25%) | 3 (25%) |  | 1.0 |
| Type IIa |  |  | 1 (25%) | 4 (33%) |  | 0.76 |
| Type IIb |  |  | 2 (50%) | 1 (8.3%) |  | 0.065 |
| Type III |  |  | 0 | 0 |  |  |
| Type IV |  |  | 0 | 1 (8.3%) |  | 0.55 |
| Type V |  |  | 0 | 3 (25%) |  | 0.27 |
|  |  |  |  |  |  |  |
| **Featured parameters** |  |  |  |  |  |  |
| CRP at diagnosis, mg/L | 63.3 [19.7-86.3] |  | 46.4 [10.3-117] | 63.3 [19.7-86.3] |  | 0.92 |
| White blood cell counts at diagnosis, /μL | 8170 [6400-10200] |  | 10000 [6968-16100] | 8030 [6400-9380] |  | 0.24 |
|  |  |  |  |  |  |  |
| - Initial treatment |  |  |  |  |  |  |
| Dose of prednisolone, mg/kg | 0.77 [0.63-0.93] |  | 0.78 [0.48-0.96] | 0.77 [0.65-0.92] |  | 0.76 |
| Immunosuppressive agents | 12 (34%) |  | 2 (50%) | 10 (32%) |  | 0.48 |
|  |  |  |  |  |  |  |

Values are presented as n (%) or median [IQR].

Mann-Whitney U test was used for comparison of continuous variables. Fisher's exact test compared categorical variables.

Abbreviations: ITAS, Indian Takayasu Clinical Activity Score; CRP, C-reactive protein; GCA, giant cell arteritis; TAK, Takayasu arteritis

| **Supplementary Table 5.** Detailed initial therapeutic regimen between the clusters | | | | |  |  |
| --- | --- | --- | --- | --- | --- | --- |
|  |  |  |  |  |  |  |
|  | Immunosuppressants-treated patients |  | Cluster 1 | Cluster 2 |  |  |
|  | (n = 30) |  | (n = 4) | (n = 26) |  |  |
| **Immunosuppressive agents** |  |  |  |  |  |  |
| Cyclophosphamide | 0 |  | 0 | 0 |  |  |
| Cyclosporine | 1 (3%) |  | 0 | 1 (4%) |  |  |
| Azathioprine | 4 (13%) |  | 1 (25%) | 3 (11%) |  |  |
| Methotrexate | 9 (30%) |  | 2 (50%) | 7 (27%) |  |  |
| Tacrolimus | 4 (13%) |  | 2 (50%) | 2 (7.7%) |  |  |
|  |  |  |  |  |  |  |
| Biologics | 8 (23%) |  | 2 (50%) | 6 (19%) |  |  |
| - Infliximab | 1 (3%) |  | 0 | 1 (4%) |  |  |
| - Tocilizumab | 7 (23%) |  | 2 (50%) | 5 (19%) |  |  |
|  |  |  |  |  |  |  |

Values are presented as n (%).

**
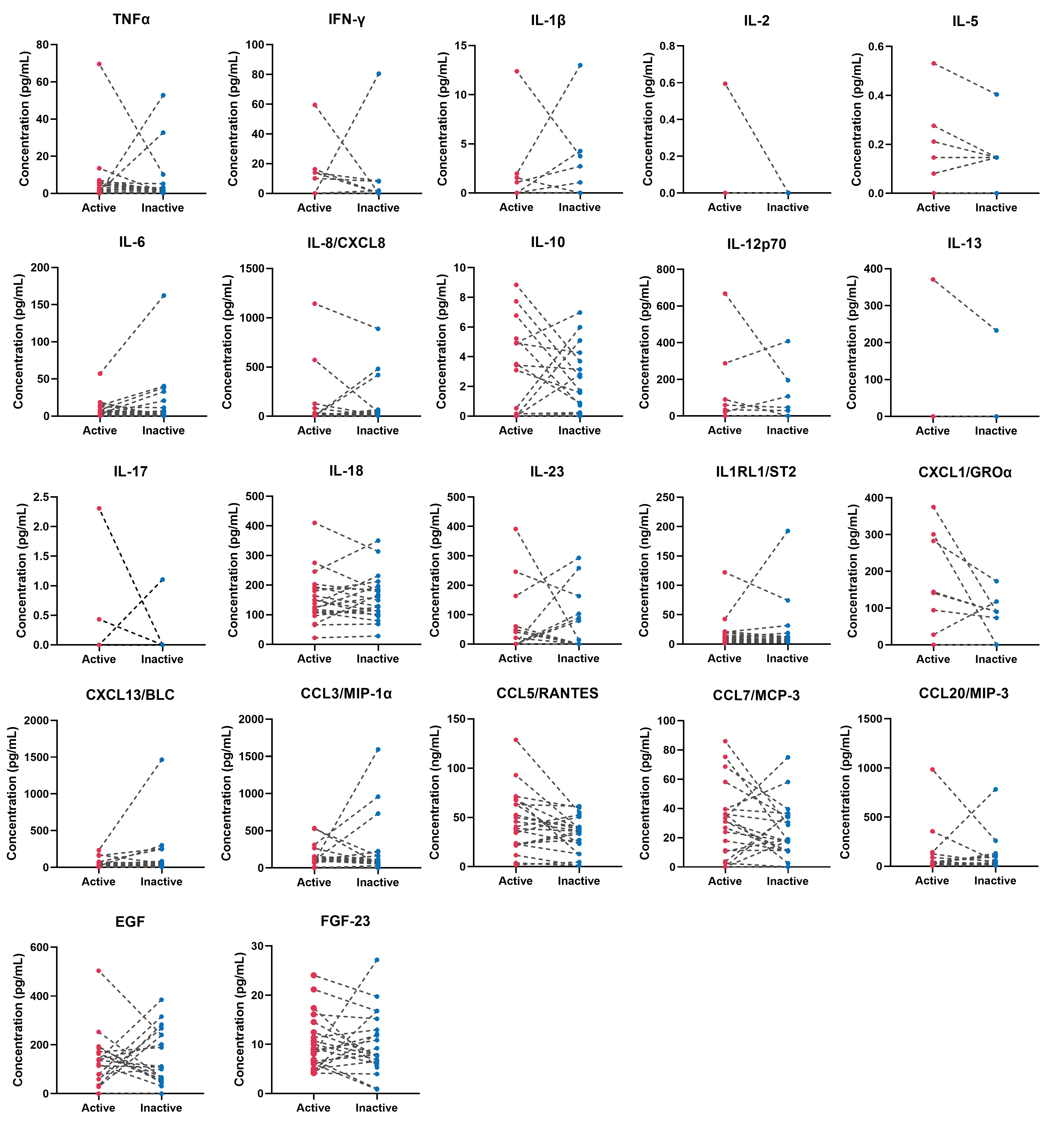
Supplementary Figure 1.** Multiplex serum cytokine/chemokine analysis results in the patients with large-vessel vasculitis (LVV). Twenty-one paired bloods from the LVV patients with active disease LVV (red) and inactive (blue) were collected and examined using multiplex cytokines/chemokines analysis including 28 analytes. A dot plot with a line shows individual cases. Wilcoxon singed-rank test for paired comparisons. Abbreviations: TNF, tumour necrosis factor; IFN, interferon; IL, interleukin; IL1RL1, IL-1 receptor-like 1; CXCL, C-X-C motif chemokine ligand; GRO, Growth-related oncogene; BLC, B lymphocyte chemoattractant; CCL, C-C motif chemokine ligand; MCP, monocyte chemotactic protein; MIP, macrophage inflammatory protein; RANTES, Regulated on Activation, Normal T cell Expressed and Secreted; EGF, epidermal growth factor; FGF, fibroblast growth factor; VEGF, vascular endothelial growth factor

**
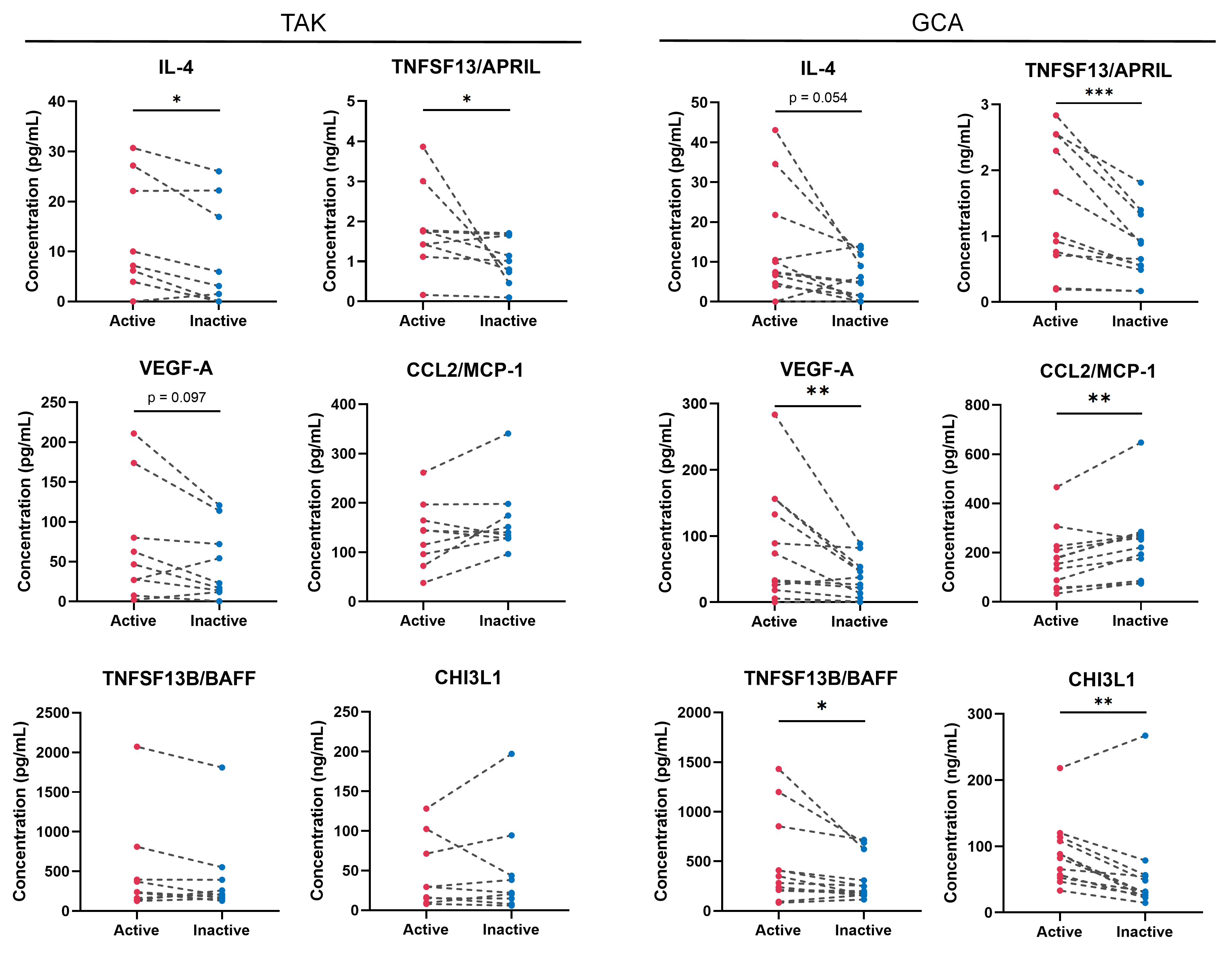
Supplementary Figure 2.** Sensitivity analysis for multiplex serum cytokine/chemokine levels in the patients with Takayasu arteritis (TAK) and giant cell arteritis (GCA). Paired blood from 9 TAK and 12 GCA patients with active disease (red) and inactive disease (blue) were collected and examined using multiplex cytokines/chemokines analysis. A dot plot with a line shows individual cases. Wilcoxon singed-rank test for paired comparisons. Abbreviations: IL, interleukin; TNFSF, tumour necrosis factor superfamily; APRIL, A proliferation-inducing ligand; VEGF, vascular endothelial growth factor; CCL, C-C motif chemokine ligand; MCP, monocyte chemotactic protein; BAFF, B cell activating factor belonging to the tumour necrosis factor family; CHI3L1, Chitinase-3-like-1.
